# Carbon Dots for the Treatment of Inflammatory Diseases: An Appraisal of *In Vitro* and *In Vivo* Studies

**DOI:** 10.1155/2023/3076119

**Published:** 2023-05-25

**Authors:** Anshul Sharma, Hyo-Kyoung Choi, Hae-Jeung Lee

**Affiliations:** ^1^College of Bionanotechnology, Department of Food and Nutrition, Gachon University, Gyeonggi-do 13120, Republic of Korea; ^2^Korea Food Research Institute, 245, Nongsaengmyeong-ro, Iseo-myeon, Wanju-gun, Jeollabuk-do, Republic of Korea 55365; ^3^Institute for Aging and Clinical Nutrition Research, Gachon University, Gyeonggi-do 13120, Republic of Korea; ^4^Department of Health Sciences and Technology, GAIHST, Gachon University, Incheon 21999, Republic of Korea

## Abstract

In recent decades, several studies demonstrating various applications of carbon dots (C-dots), including metal sensing, bioimaging, pH sensing, and antimicrobial activities, have been published. Recent developments have shifted this trend toward biomedical applications that target various biomarkers relevant to chronic diseases. However, relevant developments and research results regarding the anti-inflammatory properties of C-dots against inflammation-associated diseases have not been systematically reviewed. Hence, this review discusses the anti-inflammatory effects of C-dots in *in vivo* and *in vitro* models of LPS-induced inflammation, gout, cartilage tissue engineering, drug-induced inflammation, spinal cord injury, wound healing, liver diseases, stomach cancer, gastric ulcers, acute kidney and lung injury, psoriasis, fever or hypothermia, and bone tissue regeneration. The compiled studies demonstrate the promising potential of C-dots as anti-inflammatory agents for the development of new drugs.

## 1. Introduction

Inflammation is an ancient biological process that serves to maintain human health by acting as a natural defense mechanism in the body [1, 2]. Inflammation can be categorized into different types depending on the underlying trigger (for example, an infection) and whether it is acute or chronic. As the body's primary defense, inflammation can trigger the activation of nonimmune cells (such as fibroblasts and vascular endothelial cells) and immune cells (such as neutrophils, tissue macrophages, monocytes, mast cells, and lymphocytes), which protect the host from pathogens, infections, and toxins and promote the repair and regeneration of damaged tissue, leading to the reestablishment of cellular homeostasis [3]. However, in certain circumstances, inflammation can have negative effects by inappropriately attacking tissues within the body [2]. Acute inflammation is typically less severe and limited to a specific location in the body; however, when acute inflammation fails to resolve the issue (for example, failure to clear a pathogen), chronic inflammation can occur. Chronic inflammation can subsequently develop into an autoimmune condition via the accumulation of reactive oxygen species (ROS) and/or reactive nitrogen species (RNS), which target healthy host cells, causing sickness and leading to the loss of cellular homeostasis [4].

Previous studies have suggested that persistent oxidative stress may exacerbate localized tissue damage that could result in chronic inflammation, which in turn may contribute to several chronic human diseases [5, 6]. Examples include diabetes, neurodegenerative diseases, cancer, pulmonary diseases, ischemic heart disease, liver diseases, cardiovascular diseases, and hepatitis [4, 5, 7]. ROS production can be activated by lifestyle factors such as obesity, alcohol consumption, physical inactivity, radiation, stress, and cigarette smoking, which in turn can cause inflammation [8–10]. ROS can modulate the synthesis of various inflammatory markers, such as chemokines, cytokines, cyclooxygenase-2 (COX-2), and proinflammatory transcription factors, including nuclear factor kappa light chain enhancer of activated B-cells (NF-*κ*B), tumor necrosis factor (TNF), p53, nuclear factor erythroid 2-related factor 2 (Nrf2), activator protein 1 (AP-1), hypoxia-inducible factor 1*α* (HIF-1*α*), peroxisome proliferator-activated receptor *γ* (PPAR-*γ*), and *β*-catenin/Wnt [11].

Cells naturally have enzymatic and nonenzymatic defenses against oxidative stress, but these are not always sufficient to limit the accumulation of extremely high levels of ROS. Hence, new approaches to treating inflammation and oxidative stress-mediated diseases that simultaneously avoid excessive ROS generation and boost antioxidant defense capabilities may be effective. In this regard, the use of nanomaterials has demonstrated significant promise in reducing high ROS levels and inflammation, garnering interest as an alternative strategy for treating chronic disorders. Nanomaterials can have anti-inflammatory, antimicrobial, antioxidant, and antidiabetic effects and can be used to facilitate drug delivery, treat cardiovascular and kidney disorders, and mediate catalytic capabilities. As such, nanomaterials hold great promise in the biological and research sectors [12–15].

Nanoparticles (NPs) can be synthesized using both organic and inorganic materials. Organic NPs include polymeric NPs, poly (lactic-co-glycolic acid) (PLGA), polyvinylpyrrolidone (PVP) NPs, poly(N-(2-hydroxypropyl)methacrylamide) (PHPMA), chitosan NPs, dendrimer-based NPs (dendrimers such as polyamidoamine (PAMAM), polypropylene imine (PPI), poly (glycerol-co-succinic acid), and poly-l-lysine (PLL)), and liposomal NPs. Inorganic NPs include quantum dots, carbon NPs (single-walled and multiwalled carbon nanotubes), and iron oxide NPs [16]. Their outstanding features are their distinctive size, shape, and surface properties for tissue penetration via a passive or active targeting mechanism [17].

In this manuscript, we discuss numerous attributes of carbon dots (C-dots) pertaining to their anti-inflammatory properties, review representative studies of their anti-inflammatory activities, and offer viewpoints on the challenges in the development and application of this new class of anti-inflammatory agents.

## 2. Carbon Dots (C-dots)

C-dots were serendipitously discovered in an arc discharge shoot as a fluorescent fraction during the purification of single-walled carbon nanotubes and were identified as nanostructured carbon materials during atomic force microscopy observation [18]. Since then, the scientific community has become interested in this new carbon material because of its unique photoluminescence features, chemical stability, almost negligible toxicity, ease of synthesis, and environmental friendliness [19–21]. In particular, C-dots overcome the drawbacks of metal-based quantum dots, including photobleaching or photoblinking, toxicity, and high fabrication costs [22–24].

C-dots are a new family of quasispherical (with sp2/sp3), zero-dimensional carbon nanoparticles with diameters less than 10 nm that are known for their unique fluorescence properties [25, 26]. C-dots exhibit a core-shell structure with graphitic or amorphous carbon and different functional groups, such as amino (–NH_2_), carboxyl (–COOH), and hydroxyl (–OH) groups, in the shell, which makes them water-soluble. More significantly, their surface properties are tunable and can be changed to improve their photoluminescence, biocompatibility, and other physical and chemical properties [27–30]. Their synthetic route, size, surface properties, and composition have considerable impacts on their physiochemical, optical, and electrical properties and, consequently, their applications in the biomedical, energy, and healthcare fields [31, 32]. Carbon quantum dots (with quantum confinement and crystalline structures), graphene quantum dots (*π*-conjugated single sheets), and carbon nanodots (amorphous quasispherical) are the three types of C-dots based on their unique carbon core design and surface moieties [26, 33].

In particular, amino, carboxyl, hydroxyl, and other groups on the surface of C-dots facilitate further modifications, which improve their optical properties, biocompatibility, and targeting ability, thus enhancing their sensitivity and selectivity and expanding the range of applications [34]. The desirable qualities of C-dots for biomedical applications include adjustable photoluminescence, solubility in water, negligible cytotoxicity, high biocompatibility, biodegradability, and cost-effective synthesis. These features favor the wide application of C-dots in bioimaging (*in vitro* and *in vivo*), biological sensing, cancer therapy, drug delivery and gene transfer, photosynthesis enhancement, radioactive ion removal, seawater desalination, optoelectronic devices, catalysis, biomedicine, energy, and agriculture [35–42].

C-dots can be synthesized using either top-down or bottom-up approaches [43]. Top-down techniques turn larger materials into nanoparticles by reducing them. Top-down methods can involve aggressive oxidation agents, such as acids or voltage, and can result in higher yields. However, to fine-tune the attributes of the generated nanomaterials, lengthy synthesis times and postsynthesis processes are required [39]. Examples of top-down methods include chemical ablation [44], laser ablation [45], arc discharge [18], and electrochemical methods [46]. Using bottom-up techniques, nanoparticles can be constructed from smaller components. Morphology and size are generally easier to manage with bottom-up approaches, but synthesis takes longer and requires more effort. Examples of bottom-up methods include hydrothermal treatment [47], microwave treatment [48], solvothermal treatment [49], the reverse micelle method [50], pyrolysis [51], the template method [52], and chemical oxidation [53]. Researchers have used a variety of techniques to purify and separate compounds after synthesis, including filtering, centrifugation, dialysis, freeze drying, rotary evaporation, vacuum drying, chromatography, and ultrasonication. The majority of syntheses combine several techniques to produce pure nanoparticles [54].

**Scheme 1:**
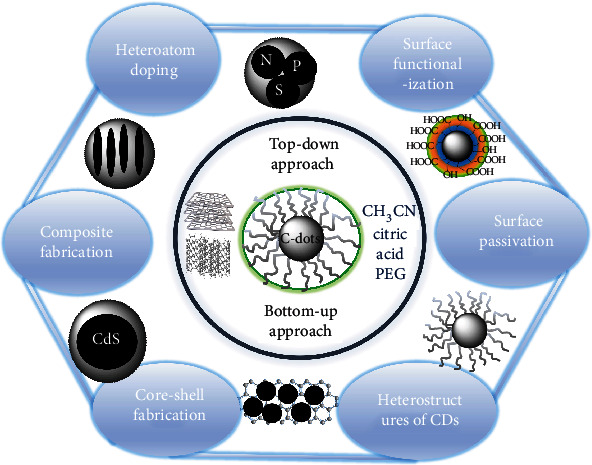
A schematic representation of the synthetic approach and surface modification of C-dots.

Although both inorganic and organic molecules can be used to prepare C-dots, over the last two decades, biomass-based materials have attracted increasing attention as starting materials for C-dot synthesis (Scheme 1). Numerous studies have discussed the significance of green C-dots [55–58]. Some examples of biomass utilized for C-dot synthesis include lotus root, coriander leaves, pumpkin, potato, lentils, garlic, starch, prawn shells, seafood waste, polysaccharides, and peach gum [59, 60]. Typically, the diameter of green C-dots ranges from 2 to 10 nm [61]. Green C-dots have many advantages over conventional C-dots, notably superior biocompatibility, the usage of building ingredients that are more environmentally friendly, and no need for heteroatom doping or chemical additives [62]. However, there remains much to explore when it comes to the structure of C-dots and their application in the treatment of inflammatory diseases, and the synthesis of homogenous C-dots with precise control over their size and surface properties remains to be developed.

## 3. *In Vitro* and *In Vivo* Studies

Recent research has revealed a direct connection between chronic diseases and inflammation; inflammation is thought to be the primary cause of chronic damage and related disorders [63]. This section sheds light on the anti-inflammatory properties of C-dots using various cell and animal disease models (Table 1).

Recently, pharmacological molecules have been used as starting materials to produce C-dots. The nonsteroidal anti-inflammatory drug aspirin has a long history of therapeutic use but is not without side effects, such as low solubility and negative effects on the stomach. Despite these side effects, current research indicates that aspirin can reduce the risk of cancer and heart disease [64, 65]. The anti-inflammatory properties of aspirin inspired Xu et al. [66] to create fluorescent aspirin-based C-dots (FACDs) for anti-inflammatory and bioimaging applications. FACDs exhibited anti-inflammatory activity *in vitro* and *in vivo* while causing minimal toxicity. Notably, at a concentration of 100 mg/mL, FACDs were found to be more anti-inflammatory than aspirin alone and reduced the expression of inflammatory markers such as TNF-*α* and IL-1*β*, *in vitro*. FACDs displayed comparable anti-inflammatory effects *in vivo* in the carrageenan-induced inflammation model, in which they effectively lowered prostaglandin E2 (PGE2) levels. No side effects caused by FACD treatment were detected in hematological analyses, and no toxicity to the liver, gallbladder, or kidney was observed, suggesting that C-dots may be used for *in vivo* applications. FACDs were also found to have good bioimaging capacity both *in vitro* and *in vivo*. The findings demonstrated that FACDs have two functionalities, cellular imaging/bioimaging and anti-inflammation, and suggested that FACDs have a high potential for future therapeutic applications [66] (Table 1).

Gout, also known as inflammatory arthritis, is characterized by the accumulation of monosodium urate (MSU) crystals as a result of high uric acid levels [67]. Although first-line treatments are available, they have several disadvantages. Consequently, novel therapeutic alternatives for the treatment of inflammatory arthritis and hyperuricemia are constantly being explored and developed. C-dots derived from *Aurantii fructus immaturus* carbonisata (also known as Zhi Shi in Chinese and isolated from citrus plants; AFIC-CDs) were synthesized, characterized, and tested *in vivo* and *in vitro* for their inhibitory effect against gout and hyperuricemia [68]. AFIC-CD treatment dramatically reduced paw pressure and volume. The levels of proinflammatory cytokines generated using MSU crystals were also reduced after treatment with AFIC-CDs. The AFIC-CDs showed negligible cytotoxicity and reduced xanthine oxidase (XOD) activity in RAW264.7 cells. The authors also discovered that AFIC-CDs reduced uric acid levels in hyperuricemic rats in a time-dependent manner. AFIC-CDs (2, 4, and 8 mg/kg) inhibited XOD activity in the liver by 18, 27, and 14%, respectively, and in the serum by 25, 31.2, and 34.6%, respectively [68]. Another study by Wang et al. discovered the antigout effects of C-dots prepared from *Puerariae lobatae Radix* using an animal model. C-dots lowered blood uric acid levels in model rats by inhibiting the activity of XOD and reducing the degree of swelling and pathological damage in gouty arthritis [69] (Table 1).

Mesenchymal stem cells (MSCs) have recently attracted considerable attention as a potential clinical therapy for a variety of diseases [70]. Many studies have demonstrated the use of C-dots and CQDs as fluorescent gene-delivery vehicles. The potential of C-dots for gene therapy in cartilage tissue engineering was recently reported, whereby a safe nanovector was created by bioconjugating C-dots with a protein cross-linker, sulfosuccinimidyl-4-(N-maleimidomethyl) cyclohexane-1-carboxylate (sulfo-SMCC), to produce effector molecules for the binding and delivery of small interfering RNA (siRNA) [71]. Tumor necrosis factor-*α* (TNF-*α*) is a proinflammatory cytokine that regulates local inflammatory processes in the joint and has an inhibitory effect on chondrogenesis [72]. The study showed that bioconjugated C-dots inhibited TNF-*α* and promoted chondrogenesis from MSCs. This strategy facilitates the effective binding and delivery of siTnf*α* to MSCs. CD-SMMC upregulates cartilage-specific markers (*Sox9*, SRY-Box transcription factor 9; *Col2a1*, type II procollagen alpha-1 chain; and *Acan*, aggrecan), which aid in cartilage regeneration by inhibiting the inflammation of MSCs. CD-SMCC showed favorable biocompatibility, low toxicity, high transfection efficiency, and excellent complexing ability with siRNA. In addition, *in vivo* exploration indicated that CD-SMCC-siTnf*α*-transfected MSCs accelerated cartilage regeneration [71] (Table 1).

Inflammatory conditions have been treated with the mulberry silkworm cocoon carbonisata (MSCC) for centuries, but despite extensive research, nothing is known about the plant's anti-inflammatory constituents or molecular mechanisms. Wang et al. [73] innovatively used three classical animal models of inflammation, namely, ear edema, vascular permeability, and sepsis induced by treatment with phlogistic agents (dimethylbenzene, acetic acid, and lipopolysaccharide), to assess the anti-inflammatory effect of MSCC-CDs. MSCC-CDs demonstrated remarkable anti-inflammatory bioactivity in an LPS-induced inflammation (sepsis) model, which could be mediated by the inhibition of TNF-*α* and IL-6 serum levels. This LPS-induced model closely resembles sepsis in humans. C-dots also reduced edema caused by xylene and vascular permeability triggered by acetic acid. This study demonstrates the potential biomedical applications of C-dots, particularly as an anti-inflammatory drug [73] (Table 1).

Nitric oxide (NO) production by macrophages has a major impact on inflammatory responses [74]. Accordingly, one study showed that molasses-derived C-dots reduced LPS-induced NO generation in RAW264.7 macrophages. Although the precise mechanistic insights are still unclear, according to the authors, the uptake and cellular tracking of C-dots have been a result of receptor- or non-receptor-mediated endocytosis based on laser scanning confocal microscopy observations [75]. Another study synthesized C-dots from carob molasses and evaluated the influence of different surface passivation agents, such as alginate (ALG), polyvinyl alcohol (PVA), and polyethylene glycol (PEG). The authors discovered that PEG or PVA inhibited IL-6 and TNF-*α* production in RAW264.7 cells, whereas ALG increased TNF-*α* production, thus potentiating the proinflammatory response. C-dots with PVA demonstrated the strongest anti-inflammatory effects. This study's finding supports the hypothesis that the presence of different functional groups affects the applicability of C-dots [76] (Table 1).

Ibuprofen is one of the most widely used nonsteroidal anti-inflammatory drugs; however, owing to its side effects (poor solubility and gastric injury), its use in clinical applications is limited [77]. Nonsteroidal anti-inflammatory drugs act against COX enzymes to reduce the production of prostaglandins [78]. Using ibuprofen as a carbon source, CQDs were synthesized by Qu et al. and used as an anti-inflammatory agent in a carrageenan-induced animal model. The cytotoxicity of the CQDs was evaluated in HeLa cells. The functional CQDs exhibit negligible cytotoxicity, high stability and solubility, and good biocompatibility. CQDs reduced carrageenan-induced PGE2 serum levels and significantly reduced the number of neutrophils. Furthermore, the CQDs are amenable to bioimaging studies, as evidenced by their strong fluorescence (for 60 min) *in vivo*. The authors advocated that the anti-inflammatory effects were plausibly due to functional groups acquired from the carbon source ibuprofen [79] (Table 1).

The relationship between oxidative stress and inflammatory reactions is well-known [80]. A growing body of research has indicated that secondary damage cascades are significantly influenced by a large increase in ROS levels in the injured spinal cord. Traumatic and nontraumatic damage can cause spinal cord injury, with trauma being the most common cause of secondary injury. C-dots are known to have both antioxidant and anti-inflammatory effects. It is hypothesized that ROS quenching can reduce inflammation and subsequent damage after a traumatic spinal cord injury [81]. In this regard, recent work by Luo et al. [82] produced selenium- (Se-) doped CQDs and evaluated them against H_2_O_2_-induced oxidative damage *in vitro* (in astrocytes and PC12 cells) and spinal cord injury *in vivo*. Se-CQDs exhibited protective effects against spinal cord damage by preventing inflammation, neuronal cell death, and demyelination. Furthermore, after treatment with Se-CQDs, improved locomotor function was observed. The authors urge further research into biosafety and the specific underlying mechanisms of Se-CODs [82] (Table 1). Another study demonstrated the use of a solvothermal approach for the synthesis of C-dots from laccaic acid as a nanozyme that showed anti-inflammatory properties against LPS-induced macrophages (RAW264.7 cells) [83]. Nanocomposite hydrogels with C-dots have attracted considerable attention because of their simple preparation methods and useful properties. In an exemplary study, Chen et al. constructed a bioadhesive, injectable, self-healing C-dot (model cargo)-containing hydrogel prepared using derivatives of alginate and chitosan (N-CBCS/A-ALG/C-dots) that enabled efficient intergel diffusion of catalytic C-dots to the target site (skin) and relieved oxidative stress at sites of inflammation by removing excess ROS [84]. Hence, these composites (polymer and C-dots) present a novel avenue for injectable drug delivery systems that may be used in future clinical applications.

TNF-*α* induction can increase ROS levels in endothelial cells [85]. Belperain et al. reported the beneficial effects of carbon nanodots on TNF-*α*-induced ROS levels and inflammatory molecules in human microvascular endothelial cells [86] (Table 1). Likewise, the modulating effects of cerium-doped carbon nanodots on inflammation during the course of wound healing have also been reported in a mouse model. As per the authors, cerium-doped carbon nanodots have the potential to be used against oxidative stress-based diseases [87]. Furthermore, the anti-inflammatory and antioxidant activities of green C-dots synthesized from *Carica papaya* leaves have recently been reported to induce membrane stabilization in hyposaline-treated human red blood cells [88]. Another recent study showed that metal-free C-dots synthesized using ethylenediamine, phenylenediamine, and ethanol (anhydrous) reduced LPS-induced inflammation in the liver of a mouse model and promoted the scavenging of hydroxyl, superoxide anion, and peroxide radicals [12]. Moreover, a separate study showed that C-dots have enzyme-like activity against oxidative damage [89]. Taken together, these studies demonstrate that reducing oxidative stress by scavenging ROS is crucial to avoiding inflammation and illnesses caused by oxidative stress.

According to previous studies, cancer is closely associated with chronic inflammation [90]. As previously mentioned, inflammation is a defense mechanism, but in some situations, it fosters an environment that is favorable for tumor cell growth, invasion, and metastasis [91]. *Nelumbinis Rhizomatis Nodus* carbonisata (NRNC), which was recently used to synthesize C-dots and as a nanomedicine against stomach cancer, was considered a safer alternative to synthetic medications because of its lack of negative side effects. The dried nodal rhizome of *Nelumbo nucifera* is a source of NRN [92]. NRN was converted into carbon (charcoal) to produce C-dots, which were then tested for their effectiveness against ethanol-induced gastric ulcers in rats. In animal models, ethanol consumption has been shown to lead to oxidative stress and inflammatory responses. Green synthetic C-dots made from NRNC exhibited no cytotoxicity toward gastric epithelial cells (GES-1). C-dot therapy significantly reduced the detrimental effects of ethanol on the gastric mucosal layer in rats, thereby preventing gastric ulcer formation. C-dots reduced the symptoms of inflammation in test animals by inhibiting the production of proinflammatory markers and lowering oxidative stress by significantly increasing the levels of antioxidant enzymes [92] (Table 1). Similarly, Hu et al. [93] synthesized C-dots using *Radix Sophorae Flavescentis* carbonisata and demonstrated their protective effects against ethanol-induced acute gastric ulcers in rats. The anti-inflammatory effects of C-dots were due to the downregulation of the NF-*κ*B pathway, which inhibited IL-6 and TNF-*α*. C-dots also upregulated the expression of enzymatic and nonenzymatic antioxidants and downregulated the levels of iNOS and the lipid peroxide metabolite malondialdehyde (MDA), suggesting antioxidant effects. The synthesized C-dots exhibited negligible toxicity and good bioavailability [93]. Similarly, another study demonstrated the antigastric ulcer effects of C-dots derived from *Glycyrrhizae Radix et Rhizoma* (GRR) [94]. GRR-C-dots minimized the detrimental effects of alcohol by alleviating oxidative stress in the mucosal layer, similar to a previous study. C-dots restored MDA and superoxide dismutase (SOD) levels. Gastric NO levels decreased significantly after C-dot treatment, although no effects were observed on serum NO levels [94]. The antigastric cancer effect of seven semicarbonized nanodots from different herbs was recently demonstrated by Lu et al. [95]. *Atractylodes macrocephala*-derived nanodots (a medicinal herb) showed excellent activity against a gastric ulcer animal model. The protective effects of carbon nanodots (CNDs) included inhibiting proinflammatory cytokine production, alleviating oxidative stress, and increasing PGE2 and mucin MUC5AC secretion to protect the gastric mucosa. The inhibition rate of the CNDs was approximately 90%. Additionally, CND treatment resulted in lower levels of both dopamine and 5-hydroxytryptamine in the brain, which lowered the neurobiological response induced by stress. The CND treatment also restored normal bacterial diversity and regulated energy metabolism. According to the researchers, the semicarbonized nature of CNDs is the primary contributor to their biological properties against stomach ulcers [95] (Table 1). These studies showed that green C-dots have antiulcer and gastroprotective properties that may be used as therapeutic candidates to treat gastric cancer.

Acute inflammatory reactions commonly occur after a sting or bite from venomous creatures (e.g., snakes) [96]. A recent study demonstrated that acute kidney injury caused by snake venom can be prevented using green C-dots synthesized from *Phellodendri chinensis* cortex [97]. The authors used lyophilized venom of *Deinagkistrodon acutus*, which is considered the most perilous snake in China, and acute kidney injury was observed as a severe systemic reaction following venom injection. A topical antidote is available for *Deinagkistrodon acutus*, but it is not without side effects. Therefore, complementary options are always considered. The use of C-dots is an effective treatment option against kidney inflammatory responses, and the authors observed protective anti-inflammatory effects in the kidneys of mice injected with *Deinagkistrodon acutus* venom, including lower expression of monocyte chemotactic protein 1 (MCP-1), IL-1*β*, and IL-10. MCP-1, a member of the chemokine family, is produced by the local kidney and inflammatory cells (Table 1) and plays a role in the activation and recruitment of leukocytes during inflammatory responses [98]. C-dots were also found to improve kidney function in the mouse model. Thus, this study demonstrated, for the first time, a novel biomedical application of C-dots against snake venom [97]. In another study, the same research team created *Phellodendri chinensis cortex*-based C-dots utilizing the calcination process and showed that they had positive effects on mice with skin conditions similar to psoriasis (a chronic inflammatory skin disorder) that were brought on by typical imiquimod. According to the authors, C-dots shifted microglial polarization from the M1 state to the M2 state. Antipsoriasis effects were observed in both cell and animal models [99] (Table 1).

The anti-inflammatory potential of heme oxygenase-1 (HO-1), a protective enzyme, has been observed following the activation of several signaling pathways. It has been suggested that increased production of HO-1 together with other antioxidative enzymes and the regulation of signaling pathways play a significant role in the prevention of acute lung injury [100]. Considering the therapeutic importance of HO-1, C-dots made from L-ascorbic acid were used to target LPS-induced acute lung damage in a mouse model. C-dot treatment resulted in upregulated expression of HO-1 and modulation of the BTB and CNC homology (BACH) signaling pathways, which led to an anti-inflammatory effect and an improved survival rate. Furthermore, lower levels of IL-6 and TNF-*α* were detected in the lung tissues of C-dot-treated mice, and enhanced HO-1 transcriptional and translational expression was observed *in vitro* [101] (Table 1). Similarly, another study demonstrated the protective effects of C-dots from *Armeniacae Semen Amarum* carbonisata against LPS-induced acute lung injury *in vivo*. C-dot treatment decreased the serum levels of proinflammatory cytokines (IL-1*β*, IL-6, and TNF-*α*) while increasing IL-10 levels. Furthermore, C-dots also demonstrated antioxidant capacity by increasing glutathione content and SOD activity. C-dots significantly lowered myeloperoxidase (MPO) activity and MDA levels in the lung tissues of the animal model, indicating mitigation of the harmful effects of inflammation and improvement of the antioxidant status [102] (Table 1). Both these studies demonstrate the clinical potential of C-dots against LPS-induced pneumonia.

The prevalence of nonalcoholic fatty liver disease (NAFLD), an umbrella term describing a range of liver disease conditions, is increasing worldwide. NAFLD can lead to another inflammatory state in the liver, known as nonalcoholic steatohepatitis [103]. In addition to inflammation, surplus iron (Fe) is believed to play a major role in the development of NAFLD [104]. Considering this, a recent study demonstrated the synthesis of C-dots capable of chelating Fe ions in zebrafish [105]. The authors used Fe and egg whites for the synthesis of C-dots, and their treatment resulted in a reduction in ROS levels, endoplasmic stress, and hepatic cell apoptosis. C-dot treatment also regulated the NF-*κ*B signaling pathway, which showed beneficial anti-inflammatory and antioxidative effects against NAFLD. The authors also stated that the Fe-chelating ability of the synthesized C-dots was comparable to that of other Fe chelators, ethylenediaminetetraacetic acid, and deferiprone. C-dots showed excellent biocompatibility *in vitro* and *in vivo* and were utilized for the real-time monitoring of Fe ions *in vivo* [105]. In addition to NAFLD, anomalous activation of the NF-*κ*B pathway can lead to other disorders. Hence, continuous efforts have been directed toward investigating NF-*κ*B inhibitors. CdTe quantum dots have shown for the first time that they can selectively block the NF-*κ*B pathway by inhibiting the activation of I*κ*B kinase alpha/beta (IKK*α*/*β*), resulting in to the clampdown of both canonical and noncanonical NF-*κ*B signaling pathways *in vitro* and *in vivo*. CQDs have shown multifactorial applications, which include anticancer and antiviral effects [106]. C-dots prepared using citric acid and glutathione demonstrated strong anti-inflammatory potential against LPS-induced inflammation in J774A.1 cells (macrophages) by regulating the NF-*κ*B signaling pathway and mitigating ROS moieties [107] (Table 1). These studies suggest that C-dots may be used as potential candidates for oxidative stress-related inflammatory disorders.

Inflammation caused by an increase in numerous proinflammatory proteins drives the pathogenesis of ulcerative colitis (UC). C-dots made from the carbonized product of *Rhei Radix et Rhizoma* reduced inflammatory reactions and oxidative stress damage by increasing IL-10, GSH, SOD, and CAT levels and decreasing IL-6, TNF-*α*, MDA, and MPO levels [108] (Table 1). Another study described the synthesis and anti-inflammatory effects of C-dots synthesized from *Lonicerae japonicae* Flos in an LPS-induced rat model with fever and hypothermia symptoms [109]. C-dots lowered body temperature and downregulated the expression of proinflammatory cytokines. The link between TNF-*α* and hypothermia has been reported previously [110]. This study indicated the anti-inflammatory effects of C-dots in dealing with fever or hypothermia [109].

Another area in which C-dots may be exploited is bone tissue regeneration. Inflammation is the main obstruction to bone regeneration and is often aggravated by increased ROS levels. Thus, inhibition of inflammation during bone regeneration is a major challenge. A recent study demonstrated the synthesis of C-dots from citric acid (5 parts), ammonium fluoride (1 part), and a small amount of dexamethasone (a drug that relieves inflammation), which showed anti-inflammatory properties and exhibited superior osteogenesis-promoting potential in both normal and inflammatory environments. The authors suggested that the anti-inflammatory activity of C-dots may be due to the presence of functional groups and the biological potential of dexamethasone. C-dots also promoted macrophage plasticity from the M1 to M2 phenotype *in vivo*, demonstrating the anti-inflammatory properties of C-dots [111] (Table 1). Similarly, another recent study demonstrated the macrophage plasticity of C-dots in an immunosuppressive sepsis mouse model. C-dots downregulated proinflammatory cytokines and upregulated anti-inflammatory cytokines. In addition, aggregated C-dots loaded into the lysosomes of macrophages demonstrated excellent antibacterial ability. Based on these findings, the multimodal activities of C-dots, including antibacterial, anti-inflammatory, and immunomodulation, offer a new approach to treating sepsis [112] (Table 1). Recently, C-dots have been used as an antifrostbite agent in a study that reported the use of *Artemisiae Argyi Folium* (AAF) carbonisata to generate C-dots. Mechanistically, C-dots improved local inflammation by reducing the expression of inflammatory mediators in mice and lowering blood glucose levels [113]. Overall, these studies demonstrate the promising potential of C-dots as anti-inflammatory agents for the development of new drugs.

## 4. Conclusions and Future Directions

This review outlined the fascinating ability of C-dots to function as anti-inflammatory agents targeting inflammation-associated diseases, demonstrating the exceptional potential of C-dots as nanomedicines. It is worth noting that research into the anti-inflammatory properties of C-dots has gained momentum in recent years. As we have summarized, C-dots have been applied to many preclinical studies involving several cell lines and animal models, which have focused on diseases such as LPS-induced inflammation, gout, cartilage tissue engineering, drug-induced inflammation, spinal cord injury, wound healing, NAFLD, stomach cancer, gastric ulcers, acute kidney and lung injury, frostbite, psoriasis, fever or hypothermia, and bone tissue regeneration. C-dots have demonstrated protective effects by lowering ROS levels, acting as antioxidant enzymes, and modulating inflammatory indicators and signaling pathways, such as NF-*κ*B cell signaling. There is no doubt that the development of C-dots with anti-inflammatory activity has made rapid progress. Future investigations of C-dots targeting other inflammation-induced pathologies and associated signaling pathways should be conducted to further realize their therapeutic potential.

C-dot research has advanced significantly in terms of synthetic techniques, structures, characteristics, mechanistic insights, and application development, but many challenges remain before their full potential can be realized. It is important to note that following their serendipitous discovery in 2004, C-dots were prepared from a variety of biomass and nonbiomass raw supplies. Moving forward, it will be worthwhile to create new C-dots with improved features and applications. The physicochemical properties of C-dots can be efficiently improved by heteroatom doping through modification of their electronic states. Future studies should concentrate on doped C-dots with enhanced anti-inflammatory characteristics, which may also be employed for other therapeutic applications. Several studies compiled in this review demonstrated the negligible cytotoxicity of C-dots against cells *in vitro*; however, the toxicity and fluorescence properties of C-dots are significantly influenced by their size, which may limit their potential use, particularly *in vivo* [114]. Thus, the routine production of large quantities of C-dots must involve extensive toxicity testing both *in vitro* and *in vivo*. Surface engineering techniques that use biocompatible polymers or different passivation agents at appropriate concentrations may yield awesome biological applications without significant toxicity and may overcome the low quantum yield (QY) issue [115]. Therefore, future research should focus on the planned and controlled synthesis of C-dots, improvements to current protocols for colloidal and storage stability, and novel specialized properties and applications. Furthermore, raw materials with good biodegradability and low immunogenicity, especially in humans, should be explored.

Batch-to-batch reproducibility is another issue; C-dots prepared in various batches frequently exhibit varying results in terms of their size, surface characteristics, and QY. This may be a consequence of the initial raw material supply, synthesis route, and reaction conditions, and it limits the use and commercialization of C-dots for biomedical applications [116]. In-depth comparisons of C-dot synthesis approaches should be the subject of future studies. To evaluate structural-functional relationships, advanced characterization techniques such as synchronous X-ray radiation, spherical-aberration correction EM matrix-assisted laser desorption ionization time-of-flight mass spectroscopy, and time-resolved electron paramagnetic resonance should be used in the near future [117]. The development of environmentally friendly mass production techniques is critical to the widespread use of C-dots [58, 118].

Routine production of C-dots leads to the release of a large number of nanomaterials, knowingly and unknowingly, into the environment. This could have adverse effects on biological entities and create environmental issues. Thus, from the perspective of safety, much more attention must be paid to this area of research [119].

Concerning novel anti-inflammatory strategies, future studies should concentrate on the anti-inflammatory capabilities of C-dot nanocomposites, as well as other biological applications [120]. For example, nanocomposites of hydrogels and C-dots have been reported to scavenge excess ROS at the inflammation site and allow real-time monitoring of C-dot release in the hydrogel [84]. In light of this, the benefits of C-dot-based drug delivery, especially as sensing and real-time tracing probes and antioxidant, anti-inflammatory, and anticancer agents, are probably based on their optical properties and adaptability concerning surface modification.

To improve drug delivery efficiency, C-dot drug carriers with a large surface area, water solubility, biocompatibility, and nontoxicity should be developed in the near future. Furthermore, C-dots synthesized from herbal medicines that mitigate inflammation and related disorders may be a good choice. These C-dots offer an advantage over those based on chemicals and biomass in that they function without drug loading and exhibit low toxicity, particularly when compared to their chemical equivalents [121]. Another important approach is to develop multifunctional C-dots by combining various strategies, such as gene therapy, chemotherapy, and phototherapy (photodynamic and photothermal), to facilitate comprehensive theranostic applications of C-dots [122]. In addition, photoacoustic (optoacoustic) imaging applications using C-dots may be developed to aid in the detection of inflammation [123, 124].

The application of bioinformatics and other computational tools may yield new insights into the intraparticle dynamics of C-dots. As demonstrated in a previous report, one can gain knowledge of the desired size, functional groups, and degree of surface functionalization of C-dots using molecular dynamic simulations. Such simulations should be used in the future to assess these unresolved parameters and improve the application of C-dots and nanocomposites [125]. In the near future, it will be critical to apply artificial intelligence technology to assess the influence of each operating step on the synthesis and large-scale manufacture of high-performance C-dots and other potential nanomaterials. Recently, the use of machine learning to direct high-quantum yield C-dots has been documented [126]. These tools can accelerate the prediction, optimization, and fabrication of C-dots, along with cost reduction. This may strengthen the potential of C-dots for use in future nanomedicine applications.

Almost all biological application studies have established cytocompatibility by using cultured cell models. Research in experimental animals has revealed favorable anti-inflammatory properties; however, long-term use of C-dots may harm experimental animals by generating inflammation, liver and kidney damage, and adverse immunological responses. Notably, the elimination of C-dots from the body and their complete assessment within the body concerning long-term toxicity (including immunogenicity and genotoxicity) remain a significant challenge for the scientific community. Zebrafish (*Danio rerio*) could be useful in future investigations of C-dot toxicity because of their small size, high egg production, *in vitro* fertilization, transparent embryos, and other advantages [127, 128].

Overall, the application of C-dots as anti-inflammatory-based treatments holds promise for future innovations in the clinic. Although the studies discussed herein only validated C-dots as anti-inflammatory biomedicines at the preclinical stage, the recapitulation of their efficacy in clinical trials will have a substantial impact on clinical practice. It is critical to conduct extensive studies on the pharmacokinetic properties, toxicology, and other relevant C-dot-based anti-inflammatory biological effects on the human body. Researchers from different areas of science, such as chemistry, physics, bio-nanotechnology, toxicology, biochemistry, environmental studies, and clinical medicine, must work together to establish the use of C-dots in biological applications. In particular, collaboration between disciplines will be required to address outstanding challenges related to the preparation of C-dots, including uniform size, reproducibility, biocompatibility, and long-term biological toxicity. In summary, the synthesis of high-quality C-dots is a significant challenge, besides the fact that it presents substantial promise as a biomedicine for the treatment of inflammatory diseases.

## Figures and Tables

**Table 1:** The anti-inflammatory activity of carbon dots assessed using *in vitro* and *in vivo* models.

Ref.	Type of material	Method of preparation	*In vitro*		*In vivo*		Biological activity
[66]	FACDs	Microwave-assisted	RAW264.7	Pretreatment with 0, 50, or 100 mg/mL FACDs for 1 h and 1 *μ*g/mL LPS for 24 and 48 h	Acute inflammation model using Wistar rats	300 *μ*L of FACDs (1 mg/mL)+1% carrageenan after 30 min of FACDs	In vitro: ↓TNF-*α*, ↓IL-1*β* In vitro: ↓PGE_2_ level ↑Cellular imaging/bioimaging
[68]	AFIC-CDs	Pyrolysis	RAW264.7	AFIC-CDs, 0.4, 0.8, and 1.6 mg/mL, 24 h	SD rats	Gout arthritis: 50 *μ*L (20 mg/mL) MSU crystal suspension Hyperuricemia i.p. injections of potassium oxonate (300 mg/kg) and oral hypoxanthine (500 mg/kg) AFIC-CDs (8, 4, and 2 mg/kg i.p.)	In vitro: ↓cytotoxicity, ↓IL-1*β*, and ↓TNF-*α* In vivo: model of gouty arthritis: ↓MSU crystal-induced inflammation, ↓paw pressure scores, and volumes, ↓IL-1*β*, and ↓TNF-*α* Hyperuricemia rat model: ↓hyperuricemia reduction, ↓inhibited XOD, ↓IL-1*β*, and ↓TNF-*α*
[69]	PLR-CDs	Improved pyrolysis method	LO2 and RAW264.7	4000, 2000, 1000, 500, 250, 125, 62.5, 31.25, 15.62, and 7.81 *μ*g/mL, 24 h	SD rats	Potassium oxonate (300 mg/kg) and hypoxanthine (500 mg/kg) after one hour, 4, 2, and 1 mg/kg C-dots, 12 and 24 h Monosodium urate	In vitro: no change in viability 7.81 to 1000 *μ*g/mL ↓Uric acid, ↓xanthine oxidase (plasma), ↓infiltration of inflammatory cells, ↓edema, and ↓synovial hyperplasia
[71]	Bioconjugated amine functionalized water-soluble CDs	Calcination at 350°C, 1 h	MSCs from the femur and tibia of rats	200 *μ*g/mL CDs, 10 *μ*L of sulfo-SMCC, and siTnf*α* at 4 *μ*g/mL. Different ratios of the CD-SMCC to siTnf*α* (2 : 1, 5 : 1, 10 : 1, 20 : 1, and 40 : 1) were used	SD rats	—	↑Cell viability, ↓TNF-*α*, ↑GAG content, ↑*Sox9*, ↑*Col2a1*, ↑*Acan*, ↑cartilage regeneration, and ↓inflammation
[73]	MSC-CDs	Modified pyrolysis	RAW264.7 cells	CDs (10000, 5000, 2500, 1250, 625, 312.5, 156.25, 17.13, 39.07, and 19.53 lg/mL), 24 h	Female C57 black mice and female Kunming mice	Acetic acid, DMB, and lipopolysaccharide MSC-CDs, 1.40, 0.70, and 0.35 mg/kg	↓Cytotoxicity, ↓IL-6, ↓TNF-*α* (serum levels), and ↓mouse ear edema
[75]	Sugar beet molasses-derived fluorescent CDs	Simple	RAW264.7	LPS (1 *μ*g/mL)+C-dots (0.13, 0.27, 0.54, 1.08, and 2.15 mg/mL), 24 h	—	—	↑Proliferation or phagocytosis of RAW264.7, ↓NO generation, ↑cell labeling, and ↑anti-inflammatory effect
[76]	Carob molasses-derived CDs	250°C for 45 min	RAW264.7	LPS (1 *μ*g/mL)+C-dots 1 mg/mL, 24 h and passivation agents (PEG, PVA, and alginate)	—	—	No change in viability C-dots _PVA_: ↓IL-6, ↓TNF-*α* (serum levels) C-dots _PVA_: strongest effect C-dots _PEG_: mild effect C-dot alginate: proinflammatory effect
[79]	ICDs	Microwave-assisted	HeLa	200 *μ*L of ICDs (0, 10, 20, 50, and 100 *μ*g/mL)	BABL/c mice	25 mg/kg, 24 h	Low cytotoxicity, ↓PGE_2_, ↓neutrophils, and anti-inflammatory
[82]	Se-CQDs	Hydrothermal	Astrocytes, PC12, and N2a	H_2_O_2_ (250 *μ*M) 6.25, 12.5, 25, 50, 100, and 200 mg/mL of Se-CQDs, 24 h	SD rats	2.5 and 10 *μ*g Se-CQDs, 8 weeks	In vitro: ↓cytotoxicity, ↓H_2_O_2_-induced cell death, and ↓ROS levels In vivo: higher BBB scores in CQD-treated groups, mild inflammatory cell infiltration, ↓demyelination of nerve fibers, ↑neuronal survival, ↓CD68-positive cells, ↓CS56 and GFAP expression, ↓caspase-3, ↓caspase-9, ↓Bax, and ↑Bcl-2
[83]	C-dots	Solvothermal	RAW264.7 cells	LPS (1 *μ*g/mL)+50, 100, 150, 200, 250, and 300 *μ*g/mL, 24 h	—	—	↓TNF-*α*, ↓IL-1*β*, and ↓IL-6
[84]	C-dots with hydrogel (N-CBCS/A-ALG/C-dots)	Pyrolysis	HELF	50, 100, 200, and 400 *μ*g/mL C-dot pretreatment, 4 h+H_2_O_2_ (800 × 10^−6^ m), 20 h	ICR mice	500 *μ*L A-ALG/CS hydrogel (with 500 *μ*L C-dots) O-ALG/AM/C-dots or O-ALG/AM/C-dots encapsulating 800 *μ*m H_2_O_2_ under the dorsal skin	↓ROS levels and ↓oxidative stress
[86]	CNDs	Microwave	HMEC-1	0.001, 0.03, 0.1, 0.6, and 1.2 mg/mL of CNDs, 6 h+TNF-*α* (10 ng/mL), 6 h	—	—	Gene expression: ↓IL-8, ↓ICAM, ↓IL-1*β*, and ↑HO-1
[87]	Ce-CNDs	Hydrothermal	*S. aureus*, 200 *μ*g/mL+UV light exposure 5 min L929 cells	Ce-CNDs (0, 12.5, 25, 50, 100, 200, 400, 800, and 1600 *μ*g/mL) for 24 h	SD rats	Ce-CNDs (50 *μ*g/mL), 20 *μ*L	↑Photodynamical antibacterial activity, ↓H_2_O_2_, ↑cell migration, ↑bioimaging, ↓inflammation, and ↑wound repair
[88]	*Carica papaya* leave-derived CDs	Sand bath	HRBC	CDs (10, 20, 30, 40, and 50 *μ*g/*μ*L)	—	—	EC_50_ (*μ*g/mL): HRBC (15.52), ↑anti-inflammatory EC_50_ (*μ*g/mL): DPPH (27.6), TAC (23.00), and ↑anti-antioxidant
[12]	Metal-free carbon dots (CDs)	Hydrothermal	RAW264.7	CDs (0, 2, 4, 6, 8, and 10 *μ*g/mL) for 24 h+LPS (1 *μ*g/mL), 24 h	ICR mice	CDs 5 and 20 mg/kg body weight (BW) per 3 days, 7 days+LPS 500 *μ*g/kg	In vitro: ↓TNF-*α*, ↓iNOS, ↓COX-2, ↓inflammation, ↓ROS, ↑POD-like activity, ↑CAT-like property, ↑SOD-like activity, and ↑OH scavenging capability In vivo: ↓ROS, ↓hepatic congestion
[92]	NRNC-CDs	Calcination	Human GES-1	NRNC-CD solutions (1000, 500, 250, 125, 62.5, and 31.25 *μ*g/mL), 24 h	Male SD rats	NRNC-CDs (5, 2.5, and 1.25 mg/kg), 7 days+absolute ethanol (5.00 mL/kg)	In vitro: negligible cytotoxicity In vivo: ↓ulcer index, ↑SOD, ↑CAT, ↑GSH, ↑GSH-Px, ↓TNF-*α*, ↓IL-6, ↓degeneration, and ↓hemorrhage
[93]	RSFC-CDs	Pyrolysis	GES-1	RSFC-CDs (1000, 500, 250, 125, 62.50, 31.25, and 15.63 *μ*g/mL), 24 h	Male SD rats	RSFC-CDs (0.0625, 0.125, and 0.25 mg/kg, p.o.), 7 days+absolute ethanol (5 mL/kg), 1 h	In vitro: negligible cytotoxicity In vivo: ↓ulcer index, ↑CAT, ↑SOD, ↑GSH-Px, ↑GSH, ↓MDA, ↓iNOS levels, ↓NF-*κ*B, ↓TNF-*α*, and ↓IL-6
[94]	GRR-CDs	Pyrolysis	RAW264.7	GRR-CDs (5000, 2500, 1250, 625, 312.5, 156.25, 78.1, 39, and 19.5 *μ*g/mL), 24 h	Male Kunming mice	10 mL/kg of 70% alcohol+GRR-C-dots (9, 6, and 3 mg/kg)	In vitro: negligible cytotoxicity In vivo: ↓ulcer index, ↑gastric cancer inhibition rate, ↓MDA, ↑SOD, and ↓gastric NO
[95]	SCNDs Atractylodes macrocephala (SCNDs-1)	Calcination	RAW264.7	7.81, 15.63, 31.25, 62.5, 125, 250, 500, 1000, and 2000 *μ*g/mL, 24 h	Kunming mice for toxicity test, SD rats (antiulcer test)	Kunming mice: 300 mg/kg body weight, 3 days SD rats: 0.1, 0.02, and 0.004 mg/mL, 5 days	In vitro: negligible cytotoxicity In vivo: ↓MDA, ↓LPO, ↓IL-1*β*, ↓TNF-*α*, ↑PGE2, ↑MUC5AC in gastric tissues, ↓5-HT, and ↓DA in brain tissues. Normalized bacterial dysbiosis
[97]	PCC-CDs	Pyrolysis	L02 hepatocyte and embryonic kidney 293T	PCC-CDs (5000, 2500, 1250, 625, 156, 78, 39, and 19.5 *μ*g/mL), 24 h	Kunming mice	8.0, 4.0, and 2.0 mg/kg+snake venom 0.15 mg/mL and 0.2 mL, twice a day	In vitro: no effect till 2500 *μ*g/mL In vivo: ↓MCP-1, ↓IL-10, ↓IL-1*β*, and inhibited the kidney malfunctioning
[99]	PCC-CDs	Calcination	RAW264.7, L02, and 293T	PCC-CDs (2500, 1250, 625, 313, 156, 78, and 39 *μ*g/mL), 24 h	Male BALB/c mice	PCC-CDs (0.22 mg/kg)	In vitro: no effect till 1250 *μ*g/mL M1 markers (RAW cells): ↓TNF-*α*, ↓IL-6, ↓NO, and ↓iNOS. M2 markers: ↑Arg-1, ↑IL-10 In vivo: improved skin and right ear appearance, ↓PASI scores (medium dose), ↓IMQ-induced psoriasis inflammation, ↓TNF-*α*, ↓IL-6, ↓iNOS, ↓IL-17A, and ↓IL-23 M2 markers: ↑Arg-1, ↑IL-10
[101]	L-ascorbic acid (CDs-1)	Electrochemical	RAW264.7, BV2, and N9	CDs (0.004, 0.008, 0.012, 0.016, and 0.020 mg/mL), 24 h+LPS (1 mg/mL), 12 h	BALB/C female mice	CDs-1 100 mg/kg, 7 days	In vitro: ↑HO-1, ↓NO, and ↓ROS In vivo: ↑survival rate, ↓infiltration of inflammatory cells, ↑HO-1, ↓BACH1, ↓TNF-*α*, and ↓IL-6
[102]	ASAC-CDs	Pyrolysis	A549	ASAC-CDs (1000, 500, 250, 125, 62.5, 31.25, 15.63, 7.81, and 3.91 *μ*g/mL), 24 h	SD rats	ASAC-CDs (3.75, 1.88, and 0.94 mg/kg), 10 days+LPS (5 mg/kg)	In vitro: inhibitory effects from 31.25 to 1000 *μ*g/mL concentration In vivo: ↓IL-6, ↓IL-1*β*, ↓TNF-*α*, ↑IL-10, ↓MPO, ↓MDA, ↑SOD, and ↑GSH
[105]	EWCDs	Microwave	BEAS-2B cells	EWCDs of different concentrations (0–1.5 mg/mL)	Zebrafish	4 mL EWCDs (0, 0.5, 1.0, and 2.0 mg/mL)	Fe^3+^ detection, bioimaging, ↓TG, ↓TCH, ↓FABP10a, ↓rbp4, ↓ROS, ↓bip, ↓perk, ↓ATF6, ↓CHOP, ↓Nrf 2, ↓NQO1, ↓gstp1, ↓IL-1*β*, ↓TNF-*α*, ↑TGF-*β*1, ↓SREBP1, ↓CD36, and ↑CPT-1
[106]	CdTe QDs	Microwave-assisted methods	Human pancreatic cancer Panc1 Raw264.7	CQDs (40, 80, 160, and 320 nM), 12 h+TNF-*α* Pretreatment with QDs515 and QDs545, 6 to 8 h prior to 20 mg/mL poly (I : C) or 40 ng/mL LPS	C57BL/6J mice	Pretreatment 0.6 and 1.2 nmol per mouse 4 h+LPS (1.6 mg/kg)	In vitro: ↓p-IkBa, ↓p-NF-*κ*B p65, ↓p-IkBa, ↓p-IKKa, ↓p-IKK*β*, ↓P52, ↓cIAP, ↓BCL-XL, and ↓c-FLIP
[107]	Citric acid and glutathione C-dots	Hydrothermal	J774A.1	LPS (1 *μ*g/mL)+C-dots 0.23 and 0.46 mg/mL, 24 h	—	—	↓ROS, ↓IL-12, ↑Arg-1, and ↓NF-*κ*B EC_50_ (mg/mL) = DPPH · :0.16, OH· : 0.26, and O2· : 0.13
[108]	*Rhei Radix et Rhizoma Carbonisata*	One-step pyrolysis	RAW264.7	CDs (1000, 500, 250, 125, 62.5, 31.25, and 15.625 *μ*g/mL), 24 h	BALB/C mice	0.23, 0.12, and 0.06 mg/kg+DSS 4%, 7 days	↓DAI score, ↓IL-6, ↓TNF-*α*, ↑IL-10, ↑SOD, ↑CAT, ↑GSH, ↓MPO, and ↓MDA
[109]	*Lonicerae japonicae* Flos	Calcination 350°C for 1 h	RAW264.7	CDs (2000, 1000, 500, 250, 125, 62.5, 31.25, 15.62, 7.81, and 3.90 *μ*g/mL), 24 h	SD rats	Fever LPS 20 *μ*g/kg, i.p. hypothermia LPS 500 *μ*g/kg+C-dots 0.18 mg/kg	↓Viability from 1000 to 2000 *μ*g/mL concentration ↓IL-6, ↓TNF-*α*, ↓PGE2, and ↓IL-1*β*
[111]	Citric acid (5 parts)+ammonium fluoride (1 part)	Hydrothermal	rBMSCs LPS (25 ng/mL)-stimulated RAW264.7	CDs (10, 20, 50, 100, 150, and 200 *μ*g/mL) for 1, 3, and 5 days	rBMSCs from SD rats	10, 20, 50, 100, 150, and 200 *μ*g/mL for 1, 3, and 5 days	In vitro: rBMSCs: negligible toxicity, ↑cell motility, ↑expressions of osteo-related proteins. ↑ALP, ↓TNF-*α*, and ↓IL-1*β* RAW264.7 cells: good biocompatibility, ↓TNF-*α*, and ↓IL-1*β* ·OH scavenging: EC_50_ (0.11 mg/mL) In vivo: no side effects, ↑M2-type macrophages, and ↓M1-type macrophages
[112]	Negative CDs+PEI	Microwave	Raw264.7 BMDM	LPS (100 ng/mL+10 *μ*g/mL C-dots)+6.25, 12.25, 25, 50, 100, and 200 *μ*g/mL, 24 h (cell viability)	C57 mice	MRSA (3 × 10^8^ CFUs per mouse, i.p. injection) or mixed MRSA (5 × 10^7^ CFUs per mouse, i.p. injection)+MREC (5 × 10^7^ CFUs per mouse, i.p. injection)+0.1 mL MCDs, 30 days	In vitro: excellent biocompatibility, ↓TNF-*α*, ↓IL-1*β*, ↓IL-4, IL-10, and ↓ROS In vivo: ↓TNF-*α*, ↓IL-1*β*, ↓IL-6, ↑IL-10, ↓M1 cells, and ↑M2 cells
[113]	AAFC-C-dots	Pyrolysis	—	—	Kunming mouse ice water bath experimental model	C-dots: low 3 mg/kg, medium 6 mg/kg, and high 12 mg/kg, 3 days	Serum level: ↓TNF-*α*: low (75.59 pg/mL), medium (57.12 pg/mL), and high dose (62.12 pg/mL) ↓IL-1*β*: low (171.25 pg/mL), medium (99.43 pg/mL), and high (73.93 pg/mL)

293T: embryonic kidney cell line; 5-HT: 5-hydroxytryptamine; A549: human lung adenocarcinoma cell line; Acan: aggrecan; AFIC-CDs: *Aurantii fructus immaturus carbonisata*-derived carbon dots; ALP: alkaline phosphatase; Arg: arginase; ASAC-CDs: *Armeniacae Semen Amarum Carbonisata*-derived CDs; ATF6: recombinant activating transcription factor 6; BACH1: BTB domain and CNC homolog 1; Bax: Bcl-2 associated X, apoptosis regulator; BBB: blood–brain barrier; Bcl-2: B-cell lymphoma 2; BCL-XL: B-cell lymphoma-extra-large; bip-heavy-chain binding protein; BV2: microglial cell line; BW: body weight; c-FLIP: cellular FLICE-inhibitory protein; CAT: catalase; CD68: cluster of differentiation 68; Ce-CNDs: cerium-doped carbon nanodots; CFUs: colony forming units; CHOP: C/EBP-homologous protein; cIAP: inhibitors of apoptosis protein; CNDs: carbon nanodots; Col2a1: type II procollagen alpha-1 chain; COX-2: cyclooxygenase-2; CPT1: carnitine palmitoyltransferase 1; DA: dopamine; DAI: disease activity index; DMB: dimethylbenzene; DPPH: 2,2-diphenyl-1-picrylhydrazyl; DSS: dextran sulfate sodium; EC_50_: half maximal effective concentration; EWCDs: egg white-based carbon dots; FABP10a: fatty acid binding protein 10a; FACDs: fluorescent aspirin-based carbon dots; GES-1: gastric epithelial cell line; GFAP: antiglial fibrillary acidic protein; GRR: *Glycyrrhizae Radix et Rhizoma*; GSH: glutathione; GSH-Px: glutathione peroxidase; GSTP1: glutathione S-transferase P1; H_2_O_2_: hydrogen peroxide; HMEC-1: human dermal microvascular endothelial cells; HELF: human embryonic lung fibroblast; HO-1: heme oxygenase 1; HRBC: human red blood cells; i.p.: intraperitoneal; ICAM: intercellular adhesion molecules; ICDs: ibuprofen-based carbon quantum dots; ICR: institute of cancer research; IL-1*β*: interleukin-1 beta; IMQ: imiquimod; iNOS: inducible nitric oxide synthase; L02: human hepatocyte; LPO: lipid peroxidation; LPS: lipopolysaccharide; MCP-1: monocyte chemoattractant protein-1; MDA: malondialdehyde; MRSA: methicillin-resistant *Staphylococcus aureus*; MSC-CDs: carbonized mulberry silkworm cocoon-derived CDs; MSCs: mesenchymal stem cells; MSU: monosodium urate; MUC5AC: mucin 5AC; N9: murine embryonic microglia cell-line; NF-*κ*B: nuclear factor kappa B; NO: nitric oxide; NQO1: NAD(P)H quinone oxidoreductase 1; Nrf2: nuclear erythroid 2-related factor 2; NRNC-CDs: *Nelumbinis Rhizomatis Nodus carbonisata* CDs; p-IKK-*α*/*β*: phosphorylated-inhibitory-*κ*B kinase-alpha/beta; p-I*κ*B*α*: phosphorylated-nuclear factor of kappa light polypeptide gene enhancer in B-cell inhibitor, alpha; p-NF-*κ*B: phosphorylated-NF-*κ*B; PASI: psoriasis area and severity index; PCC: *Phellodendri chinensis cortex* CDs; PEG: polyethylene glycol; PEI: polyethylene mine; PERK: protein kinase r-like ER kinase; PGE2: prostaglandin E2; PLR-CDs: *Puerariae lobatae Radix* CDs; POD: peroxidase; PVA: poly (vinyl alcohol); RBP4: retinol-binding protein 4; ROS: reactive oxygen species; RSFC-CDs: *Radix Sophorae Flavescentis carbonisata*-based carbon dots; SCNDs: semicarbonized nanodots; SD: Sprague Dawley; Se-CQDs: selenium-doped carbon quantum dots; siTnf*α*: silenced TNF-*α*; SOD: superoxide dismutase; Sox9: SRY-Box transcription factor 9; SREBP1: sterol regulatory element-binding protein 1; sulfo-SMCC: sulfosuccinimidyl-4-(N-maleimidomethyl) cyclohexane-1-carboxylate; TAC: total antioxidant capacity; TCH: total cholesterol; TG: triglyceride; TGF-*β*1: transforming growth factor-beta 1; TNF-*α*: tumor necrosis factor-alpha; XO: xanthine oxidase; AAFC-C-dots: *Artemisiae Argyi Folium Carbonisata*-C-dots.

## Data Availability

The data used to support the findings of this study are included within the article.
